# PPMP, a novel tubulin-depolymerizing agent against esophageal cancer in patient-derived tumor xenografts

**DOI:** 10.18632/oncotarget.9050

**Published:** 2016-04-27

**Authors:** Yuqiao Sheng, Kangdong Liu, Qiong Wu, Naomi Oi, Hanyong Chen, Kanamata Reddy, Yanan Jiang, Ke Yao, Haitao Li, Wei Li, Yi Zhang, Mohammad Saleem, Wei-Ya Ma, Ann M. Bode, Ziming Dong, Zigang Dong

**Affiliations:** ^1^ Basic Medical College, Zhengzhou University, Zhengzhou, Henan, China; ^2^ The Hormel Institute, University of Minnesota, Austin, Minnesota, USA; ^3^ The First Affiliated Hospital, Zhengzhou University, Zhengzhou, China; ^4^ China-US (Henan) Hormel Cancer Institute, ZhengZhou, Henan, China

**Keywords:** tubulin-depolymerizing agent, esophageal cancer, patient-derived tumor xenograft

## Abstract

Esophageal cancer is one of the least studied and deadliest cancers worldwide with a poor prognosis due to limited options for treatment. Chemotherapy agents such as the microtubule-targeting compounds are the mainstay of palliation for advanced esophageal cancer treatment. However, the toxicity and side effects of tubulin-binding agents (TBAs) have promoted the development of novel, more potent but less toxic TBAs. Herein, we identified 2-[4-(3,4-dimethoxyphenyl)-3-methyl-1H-pyrazol-5-yl]-5-[(2-methylprop-2-en-1-yl)oxy] phenol (PPMP) as a novel TBA for esophageal cancer treatment. PPMP markedly inhibited tubulin polymerization, and decreased viability and anchorage-independent growth of esophageal cancer cell lines, effects that were accompanied by G2/M arrest and apoptosis. Importantly, we produced patient-derived esophageal cancer xenografts to evaluate the therapeutic effect of PPMP in a setting that best mimics the clinical context in patients with esophageal cancer. Overall, we identified PPMP as a novel microtubule-destabilizing compound and as a new therapeutic agent against esophageal carcinoma.

## INTRODUCTION

Esophageal cancer is the eighth most frequent malignancy and the sixth leading cause of cancer mortality worldwide [1]. According to the latest incidence and mortality global cancer statistics, 455,800 new cases of esophageal cancer were diagnosed and 400,200 deaths occurred in 2012 [2]. The incidence of esophageal cancer is extremely high in Eastern Asia and in Eastern and Southern Africa, and particularly in northern China [2, 3]. The two main types of esophageal cancer, squamous-cell carcinoma and adenocarcinoma, account for more than 90% of all cases of esophageal cancers [4]. For advanced esophageal cancer treatment, esophagectomy or radiotherapy is associated with poor prognosis owing to relatively late stage diagnosis and early systemic dissemination of disease, and chemotherapy is the mainstay of palliation in this setting [1, 5]. In the use of single-agent or combination chemotherapy, tubulin-binding agents (TBAs), such as paclitaxel, docetaxel, vinorelbine, and vindesine, play an important role in esophageal cancer treatment [3].

In mammalian cells, microtubules are formed by polymerized α- and β-tubulin heterodimers, and are crucial for cell shape and maintenance of polarity, cell proliferation, cytokinesis, signaling, trafficking, and migration [6]. The essential role of microtubules and their dynamics in forming the mitotic spindle during cell division, which drives andmediates the replicated chromosomes from parent to offspring, has made them an important therapeutic target in cancer treatment for decades [7]. The major mechanism of TBAs’ inhibition of cell proliferation is binding to microtubules and altering microtubule dynamics during the mitotic stage of the cell cycle [8].

Each of the microtubule processes, polymerization and depolymerization, is necessary for the proper execution of the cell division machinery, thereby segregating the microtubule-targeted antimitotic drugs into two major categories [9]. One category includes the microtubule-destabilizing agents, such as the vinca alkaloids, dolastatins, and colchicine and its analogues, which inhibit microtubule polymerization. Most of these agents bind at either the vinca domain or the colchicine domain of tubulin. Another category includes the microtubule-stabilizing agents, which enhance microtubule polymerization. Most of these agents bind to the same sites or to an overlapping taxoid-binding site on β-tubulin [7]. Although the TBAs, such as the vinca alkaloids and taxanes, have been used successfully for clinical anticancer therapy, many patients with cancer eventually develop acquired resistance to these agents [10]. Moreover, the toxicity and side effects of these existing agents drive the search for novel anti-microtubule drugs that are less toxic and less likely to result in resistance.

In the current study, by utilizing computational screening and molecular docking analysis, we identified 2-[4-(3,4-dimethoxyphenyl)-3-methyl-1H-pyrazol-5-yl]-5-[(2-methylprop- 2-en-1-yl)oxy]phenol (PPMP) as a novel TBA. PPMP potently induced G2/M arrest and apoptosis and inhibited proliferation and growth of different human esophageal cancer cell lines. Most importantly, we evaluated the possible clinical use of PPMP by investigating its therapeutic effects in patient-derived xenografts (PDXs) of primary human esophageal cancer.

## RESULTS

### PPMP inhibits growth of esophageal cancer cells

**Figure 1:**
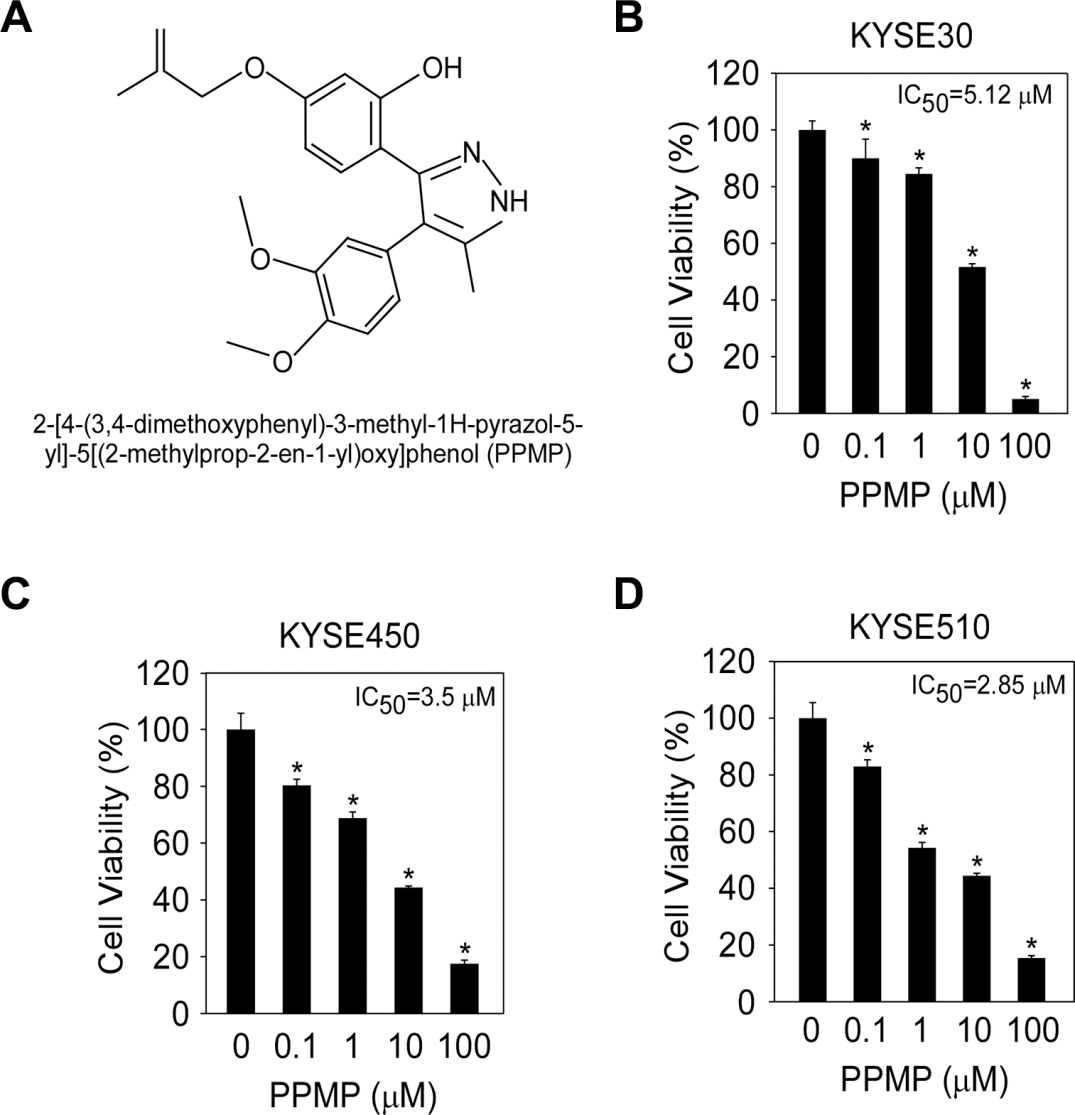
The structure of PPMP and its effect on viability of esophageal cancer cells (**A**) Chemical structure of PPMP. Viability of human esophageal cancer cells was estimated by MTS assay in (**B**) KYSE30, (**C**) KYSE450, and (**D**) KYSE510 cancer cells. All esophageal cancer cells were treated for 48 h with various concentrations of PPMP. Data are shown as mean values ± S.D. from 3 independent experiments conducted with triplicate samples. Statistical significance was determined by the Student's *t*-test (*p < 0.05 vs. untreated group).

From the computational screening results of the molecular docking analysis, we identified PPMP as a potential TBA compound (Figure 1A), which bound well with β-tubulin. To evaluate the effect of PPMP on the growth of human esophageal cancer cells, we analyzed cell viability in 3 esophageal cancer cell lines. PPMP inhibited growth of all 3 human esophageal cancer cell lines, KYSE30 (Figure 1B), KYSE450 (Figure 1C), and KYSE510 (Figure 1D), with IC_50_ values of 5.12 μM, 3.50 μM, and 2.85 μM, respectively.

### PPMP suppresses anchorage-independent growth of esophageal cancer cells

**Figure 2:**
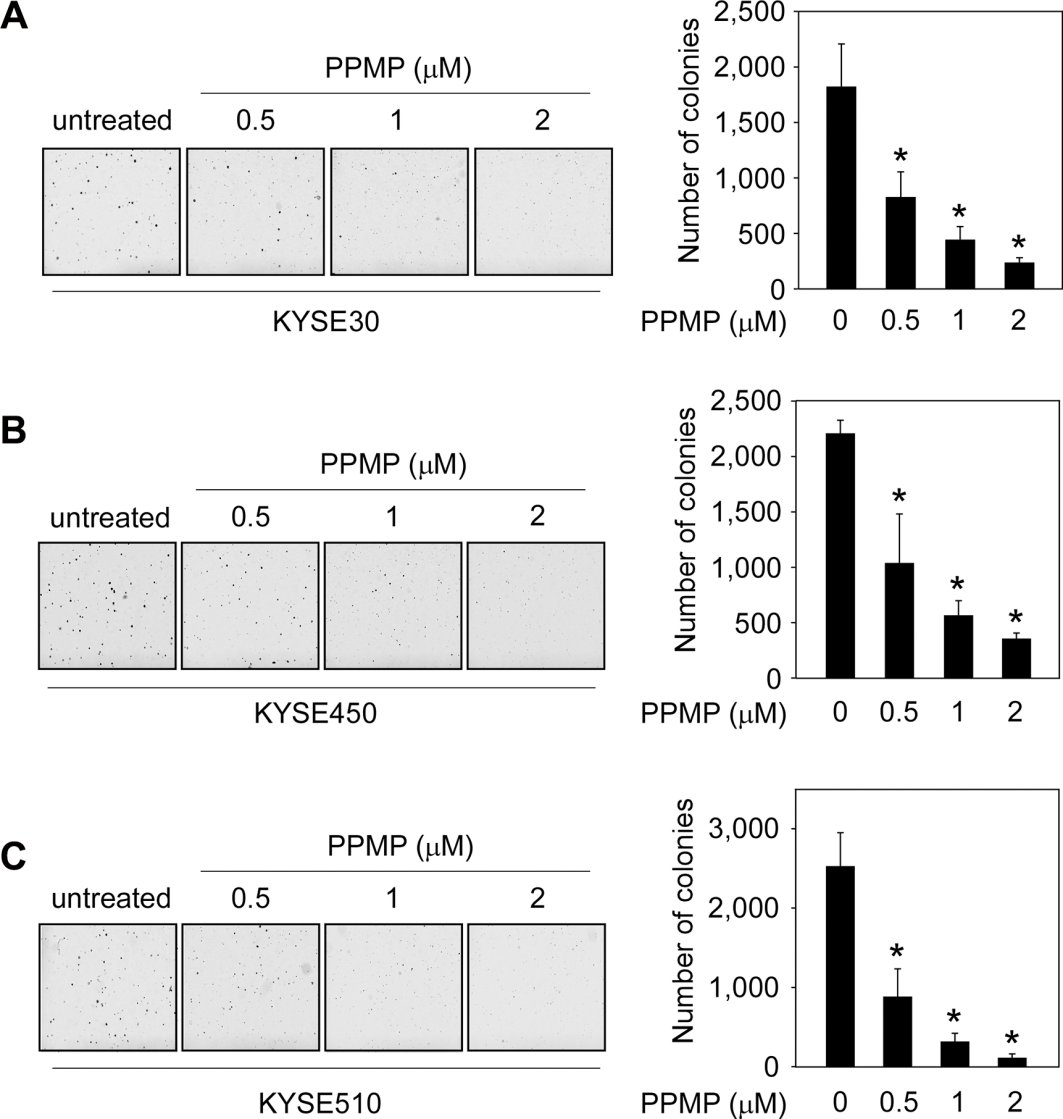
PPMP suppresses anchorage-independent soft agar growth of esophageal cancer cell lines Three human esophageal cancer cell lines, (**A**) KYSE30, (**B**) KYSE450, and (**C**) KYSE510, were treated with PPMP and assayed for their ability to proliferate in soft agar. Multicellular colony formation was photographed at 25× magnification (*p < 0.05 vs. untreated group).

To examine the effect of PPMP on anchorage-independent growth, human esophageal cancer cells were subjected to a soft agar assay. Data indicated that PPMP potently and dose-dependently, inhibited colony formation of all 3 esophageal cancer cell lines, KYSE30 (Figure 2A), KYSE450 (Figure 2B), and KYSE510 (Figure 2C), compared to untreated controls. These results show that PPMP exhibited strong antitumor efficacy against human esophageal cancer cell growth and deserves further investigation.

### PPMP triggers multinucleated cell formation and induces apoptosis in esophageal cancer cells

**Figure 3:**
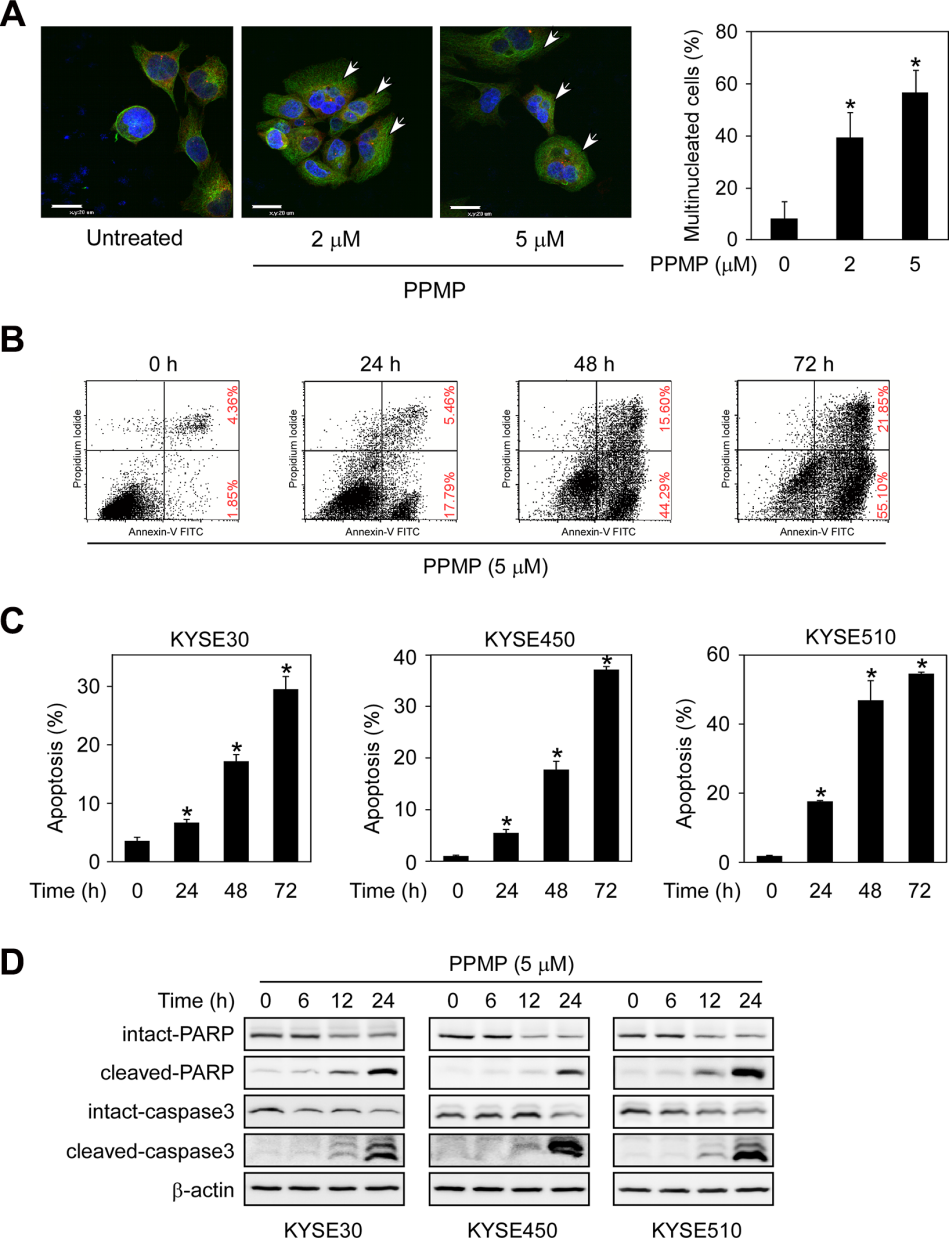
PPMP induces multinucleation and apoptosis in human esophageal cancer cells (**A**) KYSE30 cells were treated with DMSO or PPMP (2 or 5 μM) for 24 h and then stained with anti-β-tubulin (green), anti-γ-tubulin (red), and DAPI (blue). Flat cells containing multiple nuclei are indicated (arrows). Scale bar indicates 20 μm (× 600). The percentage of multinucleated cells was quantified (*p < 0.05 vs. untreated). (**B**) KYSE510 cells and each of (**C**) 3 human esophageal cancer cell lines were incubated with 5 μM PPMP for 0, 24, or 72 h. Cells were collected and apoptosis was then detected using flow cytometry and Annexin V staining (*p < 0.05 vs. 0 h). (**D**) Three esophageal cancer cell lines were treated with 5 μM PPMP and harvested at 0, 6, 12, or 24 h, and then expression of apoptotic markers was detected by immunoblotting.

To investigate the basis of PPMP's inhibitory effects, we used immunofluorescence microscopy to study the morphological phenotype of KYSE30 human esophageal cancer cells treated with PPMP. When KYSE30 cells were treated with PPMP at 2 or 5 μM for 48 h, flattened cells containing multiple nuclei were observed much more frequently compared with DMSO-treated controls (Figure 3A), suggesting a inhibition or delay in cell separation or cytokinesis. We next used Annexin V staining to measure the effect of PPMP on apoptosis in KYSE30, KYSE450, and KYSE510 cells (Figure 3B–3C). Results indicated that apoptosis was induced after exposure to PPMP (5 μM) in all 3 esophageal cancer cell lines in a time-dependent manner. The cleavage of PARP and caspase 3 facilitates cellular disassembly and are considered to be markers for cells undergoing apoptosis. Consistent with the results of flow cytometry, our Western blot resultsdemonstrated that in the presence of PPMP (5 μM), cleaved PARP or caspase 3 increasingly appeared in KYSE30, KYSE450, and KYSE510 cells over time (Figure 3D). All these data suggest that PPMP treatment potently induces multinucleation and apoptosis in human esophageal cancer cells.

### PPMP induces G2/M cell cycle arrest of esophageal cancer cells

**Figure 4:**
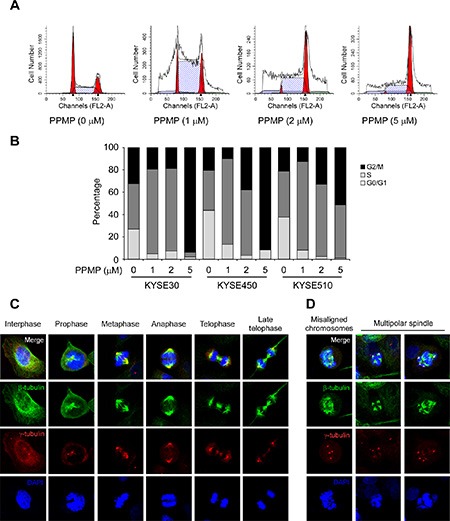
PPMP induces G2/M cell cycle arrest and affects the dynamics of microtubulin Flow cytometry analysis of cell cycle was conducted using (**A**) KYSE510 cells and each of (**B**) 3 human esophageal cancer cell lines treated for 24 h with the indicated concentrations of PPMP. Cells were harvested and cell cycle was assessed by PI staining. Interphase and mitotic KYSE30 cells treated with (**C**) DMSO or (**D**) PPMP were observed by immunofluorescence. β-Tubulin, γ-tubulin, and DNA were stained with anti-β-tubulin (green), anti-γ-tubulin (red), and DAPI (blue), respectively. Merged images are also shown at the top, where co-localization of β-tubulin and γ-tubulin results in a yellow color.

Based on the previous multinucleation and apoptosis data, we then used flow cytometry to evaluate whether PPMP influences cell cycle phase of human esophageal cancer cells. When KYSE510 cells were treated with PPMP for 24 h, we observed an increased number of cells in G2/M phase that was accompanied by a decreased cell population in the G0/G1 phase of the cell cycle, which was also observed in KYSE30 and KYSE450 cells (Figure 4A–4B). Flow cytometry results indicated that a significant G2/M arrest was induced by PPMP treatment.

Based on the total mitotic index observed in esophageal cancer cells treated with PPMP, we analyzed the microtubule stabilization and chromosomal dynamics using an immunofluorescence assay. We used KYSE30 cells, which are the largest of the 3 esophageal cancer cell lines, and used antibodies to detect β-tubulin to evaluate microtubule structure and γ-tubulin to determine the number of centrioles. DMSO-treated control cells exhibited typical cell cycle progression (Figure 4C), whereas data showed a substantial increase in aberrant mitotic structures, including misaligned chromosomes and monopolar and multipolar spindles in KYSE30 cells (Figure 4D) treated with PPMP (2 μM). These data provide evidence showing that PPMP treatment can lead to G2/M arrest in esophageal cancer cells, which is accompanied by abnormal microtubule structure and chromosome movement.

### PPMP inhibits tubulin polymerization

**Figure 5:**
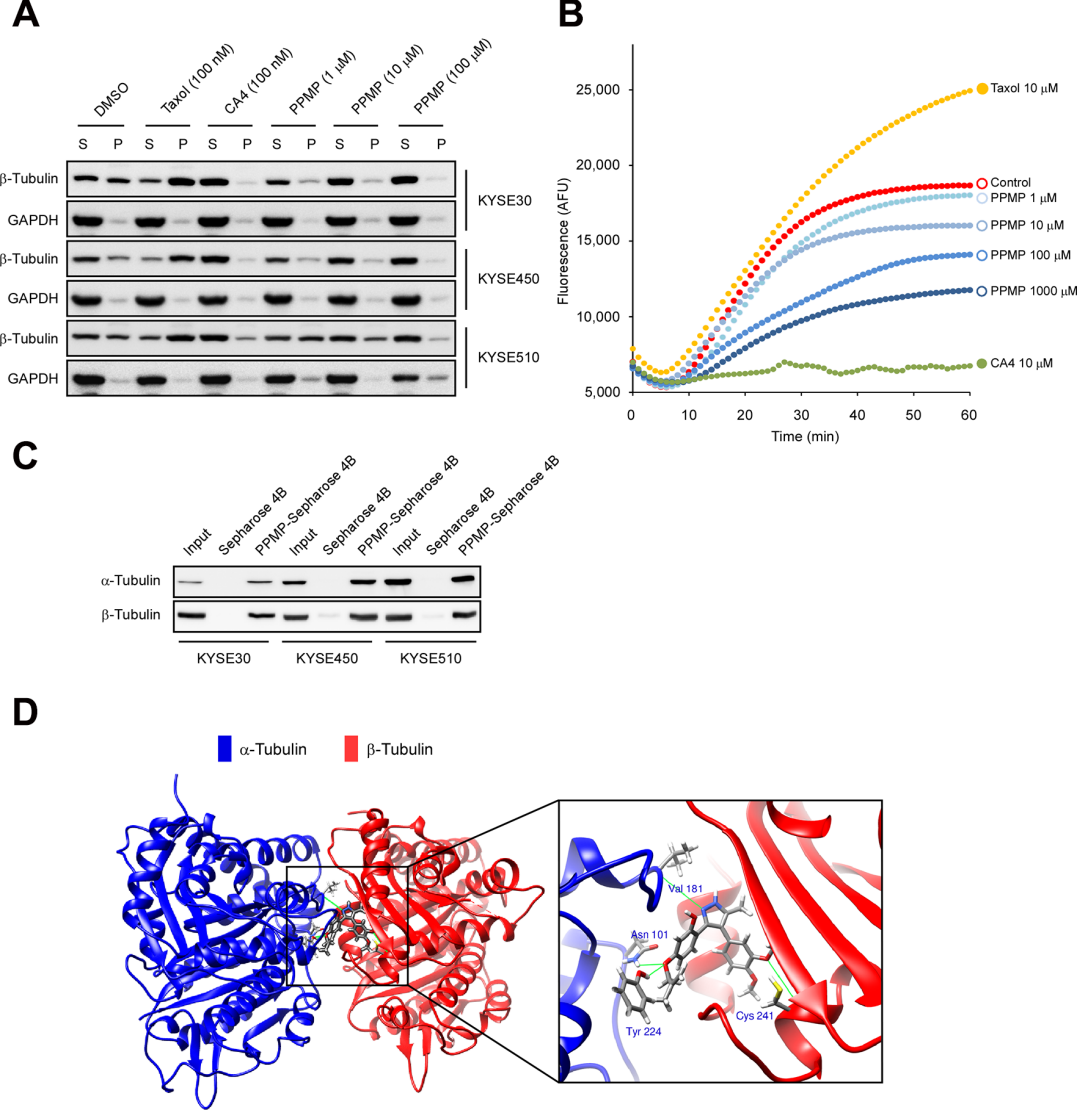
PPMP inhibits tubulin polymerization *ex vivo* and *in vitro* and also suppresses binding at the colchicine-binding site of tubulin (**A**) PPMP inhibits tubulin polymerization *ex vivo*. Top to bottom, results for KYSE30, KYSE450, and KYSE510 cells. Pellet (P) and supernatant (S) fractions containing assembled and unassembled tubulin. Tubulin polymerization as a function of the dose of PPMP in all cell lines was detectable by the increase of tubulin in the supernatant fraction and its disappearance from the pellet. (**B**) PPMP inhibits the rate of *in vitro* tubulin polymerization. The *in vitro* tubulin polymerization assay was conducted using purified porcine brain tubulin. Controls were H_2_O, 10 μM taxol, and 10 μM CA4. The results are representative of 3 independent experiments. (**C**) *Ex vivo* binding of PPMP to α-tubulin and β-tubulin was assessed by pull-down assays. Each of 3 esophageal cancer cell lysates was incubated with PPMP-Sepharose 4B beads or Sepharose 4B beads only. The protein expression was evaluated by Western blotting. (**D**) Computer docking model of PPMP and α- and β-tubulin.

To elucidate the mechanism by which PPMP affects microtubular structure in esophageal cancer cells, we focused our attention on the PPMP activity profile in *ex vivo* tubulin polymerization assays, which were performed with whole cells treated with various concentrations of PPMP or with taxol or combretastatin A4 (CA4) as positive controls. Immunoblots were performed by separating the pellet “P”, containing polymerized tubulin, and the supernatant “S” fraction, containing depolymerized tubulin. When esophageal cancer cells were treated with increasing concentrations of PPMP, the amount of polymerized tubulin was decreased, which was accompanied by an increase in depolymerized tubulin (Figure 5A). This finding suggested that PPMP treatment affects microtubular structure by enhancing tubulin depolymerization.

We then were interested in determining whether PPMP interacts with microtubules directly in cell-free tubulin polymerization assays. We performed this assay using purified tubulin cocktails with GTP at 37°C for 60 min in the presence or absence of testing drugs, including PPMP, taxol, and CA4. The data indicated that without drug treatment, tubulin subunits self-assemble to form microtubules in a time-dependent manner (Figure 5B). Similar to the addition of CA4, in the presence of PPMP, a pronounced dose-dependent reduction in microtubule assembly was observed compared with the control, whereas taxol treatment led to a dramatic increase in tubulin polymerization (Figure 5B).

To determine whether PPMP could bind directly with tubulin subunits, we conducted *ex vivo* pulldown assays using PPMP-conjugated Sepharose 4B beads. The results indicated that PPMP-conjugated Sepharose 4B beads bound to endogenous α-tubulin and β-tubulin whereas no binding was observed with Sepharose 4B beads alone (Figure 5C). Molecular docking results predicted that PPMP interacted with β-tubulin by occupying the same binding site as colchicine (Figure 5D). The computational docking model results indicated that PPMP can bind well at the colchicine binding site of tubulin and some important hydrogen bonds are formed between the compound and α- and β-tubulin (images were generated with UCSF Chimera program) [11]. This docking result shows an intermolecular interaction that provides a possible model depicting how PPMP binds with tubulin subunits and affects tubulin polymerization.

### *In vivo* antitumor efficacy of PPMP in human esophageal PDX tumor models

**Figure 6:**
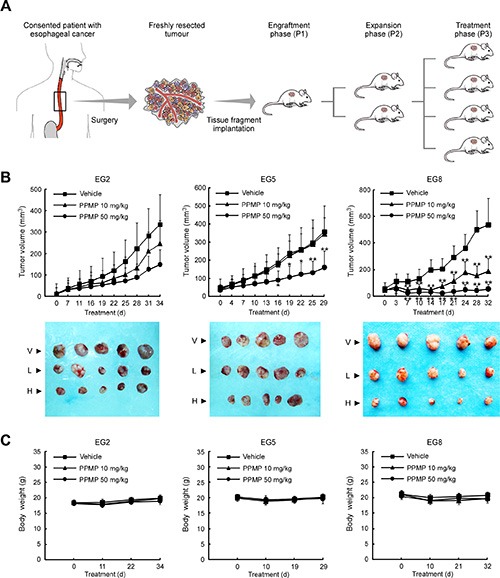
Establishment of the human esophageal patient-derived xenograft (PDX) tumor model and the antitumor efficacy of PPMP (**A**) Schematic illustration of the protocol used for generation of a human esophageal PDX tumor model (details are described in Materials and Methods). (B) PPMP significantly inhibits tumor growth in a PDX tumor model compared to a vehicle-treated group. B-17 SCID mice each implanted with a different patient's tumor were divided into 3 groups. Mice implanted with EG2 (left, *n* = 8), EG5 (middle, *n* = 7) or EG8 (right, *n* = 8) were treated every other day by i.p. injection with vehicle (▪), 10 mg/kg PPMP (▲) or 50 mg/kg PPMP (▪). Data are represented as mean values ± S.D. The asterisks indicate significant differences determined by factorial ANOVA (*p < 0.05 vs. vehicle; **p < 0.01 vs. vehicle). Representative photographs show external appearance of tumors from 3 PDX models: V, vehicle; L, 10 mg/kg PPMP; H, 50 mg/kg PPMP. (**C**) Mouse body weight changes during PPMP treatment (EG2, left; EG5, middle; EG8, right).

To test the potential benefit of PPMP treatment *in vivo*, we utilized a human esophageal PDX tumor model. Surgically resected fresh tissue fragments from three different consenting patients were subcutaneously implanted in immunodeficient mice, and then expanded to generate 3 independent xenograft tumor lines (Figure 6A). A summary of the clinical characteristics for the 3 original tumors is provided in Table 1. We selected three tumors having the same cancer grade and stage in order to maintain consistency and, thus, to facilitate statistical analysis. None of the PDX tumors received chemotherapy before surgery and subsequent implantation.

**Table 1:** Clinical characteristics of the origin used in PDX tumor models

Model ID	Gender	Age (yrs)	Source	Histology	Cancer grade	Cancer stage	Prior chemo
EG2	Male	64	Primary	ESCC	IIa	T2N0M0	No
EG5	Male	61	Primary	ESCC	IIa	T2N0M0	No
EG8	Male	63	Primary	ESCC	IIa	T2N0M0	No

ESCC: esophageal suamous-cell carcinoma.

T: tumor; N: lymph nodes; M: metastasis.

After the original tumor specimen was serially passaged to treatment phase 3 (P3), vehicle or different doses of PPMP was administered by intraperitoneal injection. Notably, at the end of the study, case EG8 was substantially more sensitive to either a high or low dose of PPMP treatment (tumor volume variation of 50 mg/kg PPMP treatment group vs. vehicle: −89.9%; 10 mg/kg PPMP vs. vehicle: −56.3%; Figure 6B). For case EG5, only the high dose of PPMP produced an obvious reduction in tumor volume (−55.1%). Case EG2 tended to respond to PPMP treatment because tumor growth appeared to be delayed, but was not statistically significant. Importantly, no significant differences in body weights occurred among the three xenograft groups treated or not treated with PPMP (Figure 6C). All these *in vivo* data indicate that PPMP treatment provides positive efficacy without causing systemic toxicity.

## DISCUSSION

Because microtubules play an extremely important role in the process of mitosis, anti-microtubule agents, like vinca alkaloids and taxanes, have been used as an effective chemotherapeutic strategy for esophageal cancer treatment over the past several decades [12, 13]. Because of the success of anti-microtubule agents in cancer therapy, developing novel agents directed against microtubules has become the focus, even as more selective approaches emerge [14]. Nonetheless, acquired resistance, significant toxicity and adverse side effects associated with existing TBAs reduce their efficacy [15, 16]. Thus, in this context, identification of novel anti-microtubule drugs with enhanced cytotoxicity against tumor cells, limited toxicity to normal tissue and insensitivity to resistance mechanisms was our intent.

In the present study, we report the discovery of a novel microtubule-destabilizing compound referred to as PPMP. The result of *ex vivo* and *in vitro* tubulin polymerization assays clearly showed that PPMP markedly inhibited tubulin polymerization in a dose-dependent manner (Figure 5A–5B). Furthermore, our *in vitro* binding assay and computer modeling results predicted a model of the interaction of PPMP with α- and β-tubulin. PPMP binds with α- and β-tubulin at the same binding domain as colchicine (Figure 5C–5D). All these results indicate that PPMP is a novel microtubule-destabilizing compound. Cells treated with anti-microtubule agents usually exhibit G2/M arrest and apoptosis [17]. We examined the effect of PPMP on human esophageal cancer cells and results showed that PPMP markedly inhibited cell viability and anchorage-independent growth of all 3 esophageal cancer cell lines studied (Figures 1, 2). The inhibition was associated with formation of multinucleated cells, accumulation of cells in the G2/M phase, and induction of apoptosis (Figures 3, 4). Similar to most TBAs that cause cancer cell death by driving them to apoptosis, necrosis or senescence, we postulated that PPMP treatment induces esophageal cancer cell death as a consequence of mitotic catastrophe [18, 19].

For *in vivo* system studies, the PDX model might be the most appropriate cancer model for studying the efficacy of treatment because it is more likely to reflect what happens in the clinic [20]. In contrast to xenografts generated from established cell lines or the genetically engineered mouse model, the PDX platform does not lead to genetic drift or is more genetically heterogeneous [21, 22]. In this study, we characterized the tumor growth responses to PPMP treatment of 3 different orthotopic esophageal cancer patient derived xenograft models for up to 34 days. Data show that PPMP effectively suppressed tumor growth without affecting mouse body weight (Figure 6). Notably, even though each of the 3 patients appears to exhibit similar clinical characteristics (Table 1), the sensitivity of each PDX tumor to PPMP was dissimilar. Therefore, further investigation of tumor cell heterogeneity, genetic background and tumor microenvironment of the original tumor will yield more specific strategies for clinical application of PPMP.

Currently, colchicine binding site inhibitors (CBSI) have been extensively studied. However, such agents have not yet reached the commercial phase for cancer therapy. We are eager to find a better CBSI having potential in clinical practice. Moreover, according to the literature, CBSIs like combretastatins and N-acetylcolchicinol-O-phosphate, have undergone considerable development as vascular-disrupting agents (VDAs) [23, 24]. The vasculature inside a solid tumor should be a superb therapeutic target. VDAs collapse the vascular structure, depriving the tumor of nutrients and oxygen that are needed for the tumor to survive [25]. As a potential CBSI, PPMP exerts its potent effects on the microtubule cytoskeleton and therefore might also produce rapid disruption of tumor blood flow. To improve our knowledge of this drug, further studies will be required to determine whether PPMP acts as a VDA or has additional targets in esophageal cancer or other cancer types.

Collectively, we provided evidence showing that PPMP suppresses the tumor growth of orthotopic PDX human esophageal tumors, a model that best mimics the clinical context. In conclusion, our *in vitro* studies and preclinical platform identified PPMP as a novel microtubule depolymerize and mitotic blocker for the chemotherapy of esophageal cancer.

## MATERIALS AND METHODS

### Chemicals and reagents

We synthesized PPMP in-house by consulting a reported protocol and adding some modification [26]. Taxol was purchased from Selleck Chemicals (Houston, TX), and combretastatin A4 (CA4) was from Sigma-Aldrich Co. (St. Louis, MO). Basal Medium Eagle (BME), L-glutamine, gentamicin, penicillin, Eagle's Minimum Essential Medium (MEM), F-12K medium, and RPMI-1640 medium were all from Life Technologies, Inc. (Grand Island, NY). Fetal bovine serum (FBS) was obtained from Gemini Bio-Products (West Sacramento, CA). Primary antibodies against α-tubulin, β-actin or GAPDH were from Santa Cruz Biotechnology, Inc. (Santa Cruz, CA) and the other antibodies were purchased from Cell Signaling Technology (Danvers, MA) unless otherwise specified. CNBr-activated Sepharose^™^ 4B beads were from GE Healthcare Biosciences (Pittsburgh, PA).

### Cell culture

The KYSE30, KYSE450 and KYSE510 human esophageal cancer cell lines were from ATCC. All cells were cultured with antibiotics at 37°C in a humidified 5% CO_2_ incubator and maintained for a maximum of 2 months (10 passages). Cells were cytogenetically tested and authenticated before freezing. All cell lines were cultured in 45% F-12K medium/45% RPMI-1640 medium supplemented with 10% FBS.

### MTS assay

Esophageal cancer cells (1 × 10^3^ cells per well) were seeded onto 96-well plates and treated or not treated with different concentrations of PPMP for measuring proliferation. After incubation for 48 h, 20 μl of CellTiter96 Aqueous MTS reagent (Promega Corporation, Madison, WI) were added to each well and then cells were incubated for 90 min at 37°C in a 5% CO_2_ incubator. The optical density (OD) was measured at 490 nm.

### Anchorage-independent cell growth assay

Esophageal cancer cells (8 × 10^3^ cells per well) were suspended in a top layer of BME/10% FBS/0.33% agar with various concentrations of PPMP (0, 0.5, 1 or 2 μM) and plated on a bottom layer of BME/10% FBS/0.5% agar with the indicated concentrations of PPMP in each well of six-well plates. After incubation for 1 to 2 weeks at 37°C in a 5% CO_2_ incubator, colonies were counted under a microscope using the Image-Pro Plus software (v.6) program (Media Cybernetics. Rockville, MD).

### Flow cytometry for analysis of apoptosis and cell cycle

For analysis of apoptosis, cancer cells (2 × 10^5^ cells per well) were seeded into six-well plates and cultured for 24 h, then exposed to 5 μM PPMP for 0, 24, 48 or 72 h. Cells were trypsinized and washed twice with cold PBS and then resuspended with phosphate-buffered saline and incubated for 5 min at room temperature with annexin V-FITC plus propidium iodide. Cells were analyzed using a FACSCalibur flow cytometer (BD Biosciences, San Jose, CA). For cell cycle analysis, cancer cells (2 × 10^5^ cells per well) were plated in 60-mm plates and cultured for 24 h, then exposed to 0, 1, 2 or 5 μM PPMP for 24 h. Cells were harvested and washed twice with PBS and fixed with cold 70% ethanol overnight at −20°C. Stained cells were detected and quantified using a FACSort flow cytometer (BD Biosciences, San Jose, CA).

### Western blot

Sample protein concentration was determined using a protein assay kit (Bio-Rad Laboratories, Inc. Hercules, CA). Total proteins (20 to 100 μg) were separated by SDS-PAGE and transferred onto a polyvinylidene difluoride (PVDF) membrane (Millipore, Billerica, MA). After blocking in 5% milk, the membranes were probed with specific primary antibodies overnight at 4°C, washed 3 times with TBS-Tween 20, and then incubated with a horseradish peroxidase (HRP) conjugated secondary antibody at room temperature 1 h for hybridization. The protein bands were visualized with a chemiluminescence reagent (GE Healthcare Biosciences, Pittsburgh, PA).

### Immunofluorescence assay

This study was conducted on KYSE30 cells in 4-chamber slides. After treatment with dimethyl sulfoxide (DMSO) or PPMP (2 or 5 μM) for 24 h, an asynchronous population of cancer cells were washed with PBS, fixed with 4% formaldehyde for 10 min, followed by blocking with 10% FBS/PBS (v/v) for 20 min. Cells were then incubated with primary antibodies, including β-tubulin rabbit mAb (1:100) or γ-tubulin mouse mAb (1:200) overnight at 4°C. After washing with PBS, secondary antibodies, including Alexa Fluor 488 rabbit IgG conjugate or Alexa Fluor 594 mouse IgG conjugate were applied. Nuclei were then demarcated using 4′,6-diamidino-2-phenylindole (DAPI, Pierce Biotechnology, Inc.) for 30 min at room temperature. Samples were evaluated by a fluorescence microscope system (Leica, Mannheim, Germany).

### Tubulin polymerization assay *ex vivo* and *in vitro*

For the *ex vivo* tubulin polymerization assay, tubulin depolymerization was measured using a modified version of a method originally documented by Minotti, *et al.* [27]. Briefly, esophageal cancer cells were grown in 60-mm plates in the presence or absence of the indicated concentrations of drugs for 24 h. Then the cells were pelleted, washed twice with PBS, and disrupted with 100 μl hypotonic buffer [0.5% NP40, 2 mM EGTA, 1 mM MgCl_2_, 20 mM Tris-HCl (pH 6.8), and a protease inhibitor mixture] for 10 min at room temperature. Lysates were then centrifuged at 13,000 rpm for 15 min at 4°C, and the soluble fraction containing depolymerized tubulin was separated from the insoluble fraction containing polymerized tubulin. Each fraction was mixed with equal volumes of 6 × SDS loading buffer, heated for 10 min at 95°C, and analyzed by Western blot. For the *in vitro* tubulin polymerization assay, performance was monitored using a tubulin polymerization kit (Cytoskeleton, Denver, CO). Purified porcine brain tubulin was resuspended on ice in ice-cold buffer [2 mM MgCl_2_, 0.5 mM EGTA, 0.5 mM EGTA, 2 mM MgCl_2_, 1 mM GTP, 15% glycerol, and 80 mM PIPES (pH 6.9)]. The 100 μL suspension was then aliquoted into a half-area 96-well plate with 5 μL of the indicated concentrations of different drugs at 37°C. Fluorescence intensity was determined by excitation at 355 nm and emission at 460 nm every min for 1 h using the Synergy 2 Multi-Mode Reader (BioTek Instruments, Winooski, VT)

### Preparation of PPMP-conjugated sepharose 4B beads

PPMP-conjugated Sepharose 4B beads were prepared following the protocol provided by GE Healthcare Biosciences. Briefly, Sepharose 4B dried powder (0.3 g) was suspended in 1 mM HCl and a 40% DMSO/H_2_O (v/v)-coupled solution [0.1 mol/L NaHCO_3_ (pH 8.3) and 0.5 M NaCl] was then mixed with compound PPMP (1.5 mg) and rotatedat 4°C overnight. The beads were transferred to 0.1 M Tris-HCl buffer (pH 8.0) and again rotated overnight at 4°C. Finally, the beads were washed with 0.1 M acetate buffer (pH 4.0) containing 0.5 M NaCl three times followed by washing once with 0.1 M Tris-HCl (pH 8.0) containing 0.5 M NaCl.

### Pulldown assays

PPMP-conjugated Sepharose 4B beads or Sepharose 4B beads only (100 μl, 50% slurry) were incubated with a KYSE30, KYSE450, or KYSE510 cell lysate (500 μg) in reaction buffer [5 mM EDTA, 1 mM DTT, 150 mM NaCl, 0.01% NP40, 50 mM Tris-HCl (pH 7.5), 0.02 mM phenylmethylsulfonyl fluoride, 2 mg/ml bovine serum albumin, and a protease inhibitor mixture] with gentle rocking overnight at 4°C. After incubation, the beads were washed 5 times with washing buffer [5 mM EDTA, 1 mM DTT, 150 mM NaCl, 0.01% NP40, 50 mM Tris-HCl (pH 7.5)], and proteins bound to the beads were boiled and analyzed by Western blotting.

### Computer modeling

To identify a new TBA and to study the interaction of candidate compounds with tubulin, we performed an extensive molecular docking analysis. First the tubulin X-ray crystal structure with a resolution of 2.30 Å [28] was downloaded from the PDB Bank [29]. Then the structure was prepared under the standard procedure of Protein Preparation Wizard in Schrödinger Suite 2014 [30]. Hydrogen atoms were added consistent with a pH of 7 and all water molecules were removed. Finally, a receptor grid of tubulin was generated based on the colchicine-binding site for studying docking. After PPMP was chosen as the candidate TBA, it was prepared under the program of LigPrep of Schrödinger Suite 2014 by default parameters for screening. Docking was accomplished using the program Glide by default parameters under the extra precision (XP) mode. Herein, we can get the best-docked representative structures.

### Patient sample selection and annotation

Human tissue collection and use protocols were approved by the ethics committee of Zhengzhou University, Zhengzhou, Henan, China. Esophageal tumor samples were obtained from 3 patients who were informed and provided written consent. Patients were treated with surgery at the First Affiliated Hospital of Zhengzhou University (Zhengzhou, Henan, China). Pathologic and clinical data were entered and maintained in our prospective database.

### PDX establishment

Based on the guidelines approved by the ethics committee of Zhengzhou University, fresh tumor tissue fragments were collected and transferred at 4°C in FBS-free RPMI-1640 medium with antibiotics. Within 2 h of surgical resection, tumor tissues were trimmed, cut into 3–5 mm sizes and implanted subcutaneously in anesthetized 6 to 8 week old female C.B-17 severe combined immunodeficient (SCID) mice (Vital River Laboratories Co., Ltd., Beijing, China). Once mass formation reached about 1500 mm^3^, mice of this first generation of xenografts (named P1) were sacrificed and the tumors were passaged and expanded for 2 more generations (named P2 and P3). When P3 tumors reached an average volume of 50 mm^3^, mice were divided into 3 groups (*n* = 7–8 mice per group) and treated with vehicle, 10 or 50 mg/kg PPMP, respectively, every other day by intraperitoneal (i.p.) injection. PPMP was dissolved in dimethyl sulfoxide (DMSO; 5%) and polyethylene glycol (PEG400; 5%) in PBS. Tumor volume [length × width × height × 0.52] and body weight were recorded twice a week.

### Statistical analysis

As necessary, all quantitative data are expressed as mean values ± standard deviation (S.D.) or standard error (S.E.). The Student's *t*-test or one-way ANOVA was conducted to determine statistically significant differences. A probability value of *p* < 0.05 was used as the criterion for statistical significance.
